# Analysis of the *agrotis segetum* pheromone gland transcriptome in the light of Sex pheromone biosynthesis

**DOI:** 10.1186/s12864-015-1909-2

**Published:** 2015-09-18

**Authors:** Bao-Jian Ding, Christer Löfstedt

**Affiliations:** Pheromone Group, Department of Biology, Lund University, Sölvegatan 37, SE-223 62 Lund, Sweden

**Keywords:** *Agrotis segetum*, Pheromone biosynthesis, Transcriptome, Desaturase, Fatty-acyl reductase, Acetyltransferase, Functional assay, Yeast expression

## Abstract

**Background:**

Moths rely heavily on pheromone communication for mate finding. The pheromone components of most moths are modified from the products of normal fatty acid metabolism by a set of tissue-specific enzymes. The turnip moth, *Agrotis segetum* uses a series of homologous fatty-alcohol acetate esters ((*Z*)-5-decenyl, (*Z*)-7-dodecenyl, and (*Z*)-9 tetradecenyl acetate) as its sex pheromone components. The ratio of the components differs between populations, making this species an interesting subject for studies of the enzymes involved in the biosynthetic pathway and their influence on sex pheromone variation.

**Results:**

Illumina sequencing and comparative analysis of the transcriptomes of the pheromone gland and abdominal epidermal tissue, enabled us to identify genes coding for putative key enzymes involved in the pheromone biosynthetic pathway, such as fatty acid synthase, β-oxidation enzymes, fatty-acyl desaturases (FAD), fatty-acyl reductases (FAR), and acetyltransferases. We functionally assayed the previously identified ∆11-desaturase [GenBank: ES583599, JX679209] and FAR [GenBank: JX679210] and candidate acetyltransferases (34 genes) by heterologous expression in yeast. The functional assay confirmed that the ∆11-desaturase interacts with palmitate and produces (*Z*)-11-hexadecenoate, which is the common unsaturated precursor of three homologous pheromone component acetates produced by subsequent chain-shortening, reduction and acetylation. Much lower, but still visible, activity on 14C and 12C saturated acids may account for minor pheromone compounds previously observed in the pheromone gland. The FAR characterized can operate on various unsaturated fatty acids that are the immediate acyl precursors of the different *A. segetum* pheromone components. None of the putative acetyltransferases that we expressed heterologously did acetylate any of the fatty alcohols tested as substrates.

**Conclusions:**

The massive sequencing technology generates enormous amounts of candidate genes potentially involved in pheromone biosynthesis but testing their function by heterologous expression or gene silencing is a bottleneck. We confirmed the function of a previously identified desaturase gene and a fatty-acyl reductase gene by heterologous expression, but the acetyltransferase postulated to be involved in pheromone biosynthesis remains illusive, in spite of 34 candidates being assayed. We also generated lists of gene candidates that may be useful for characterizing the acetyl-CoA carboxylase, fatty acid synthetase and β-oxidation enzymes.

## Background

Moths rely heavily on sex pheromones for mate finding [1]. Usually the females produce and emit the pheromone from a specialized structure, the sex pheromone gland located at the intersegmental membrane between the 8^th^ and 9^th^ abdominal segment, associated with the ovipositor at the end of the adult female abdomen [2]. Over the last five decades, sex pheromones have been identified from more than 700 species [3, 4]. The pheromone compounds are mostly fatty acid derivatives, with carbon chain length C10-C18, with 0–4 double bonds [3], and an oxygenated functional group (alcohol, aldehyde, acetate ester) [3–5]. A substantial portion is made up by acetate esters [3]. Most moths use a combination of two or more compounds in a specific ratio, constituting a more or less species-specific blend. The pheromone biosynthesis pathways have been studied extensively and are well documented in many moth species [6, 7]. Characterization of the enzymes involved in the process of pheromone biosynthesis not only helps to understand the evolution of sexual communication and speciation, but could ultimately also aid in pest control by allowing the design of drugs that block the biosynthetic machinery or by allowing the synthetic biologist to produce species-specific pheromones for mass trapping or mating disruption in biological systems like cell factories or genetically modified plants [8–10].

During the last two decades, desaturases introducing double bonds into the acyl chain in ∆6 [11], ∆9 [12, 13], ∆10 [14], ∆11 [12, 13, 15–17] and ∆14 [18] position have been cloned from many moth species, and their functions have been characterized in various heterologous expression systems. Also an omega-desaturase that introduces a double bond in the methyl terminal carbon has been cloned and characterized [19]. In some cases, two double bonds can be produced by one desaturase [17] or by the consecutive activities of two desaturases [11]. These studies highlight desaturases as key players for the diversity of pheromone structures in moth species and their role in reproductive isolation and speciation. A variety of desaturases in combination with limited chain-shortening or chain-elongation account for the diversity of double bond isomerism observed among moth sex pheromone compounds.

After the double bonds are in place and the acyl chain length is adjusted, the carbonyl carbon is modified to form a functional group. Firstly it requires a step that converts the fatty-acyl precursors into fatty alcohols. Great progress has been made since the first fatty-acyl reductase gene was identified in *Bombyx mori* [20]. Several *Ostrinia* spp. FARs have been characterized [21, 22]. The *Ostrinia* FARs are very essential to determine the final pheromone compositions [22], minimal changes in sequence can cause the pheromone component ratio to shift dramatically [23]. On the other hand, there are also FARs that are very versatile in terms of substrate specificity [24, 25], involved in the biosynthesis of multicomponent pheromones.

Fatty alcohols serve as the actual pheromone components in a number of moth species [3], but mostly, fatty alcohols will be either oxidized to aldehydes [26–28] or esterified to form acetate esters [29–31]. Acetyltransferases are important enzymes since the acetate esters are very commonly occurring pheromone components among moth species. They have not been cloned from insects yet, but have been investigated in some cases by *in vivo* labeling studies [32–34]. Unlike FARs, these functional group modification enzymes have not been studied extensively.

The chain-shortening pathway has not been characterized at the enzymatic level in insects, but it was noted in a couple of cases that mutations in the β-oxidation pathway did affect the final pheromone compositions. The major pheromone component of cabbage looper moth *Trichoplusia ni* is Z7-12:OAc, whereas a mutant strain produced a greatly increased amount of Z9-14:OAc [35, 36].

The turnip moth, *A. segetum* uses a series of short chain acetate esters as sex pheromone components, including the homologues (*Z*)-5-decenyl, (*Z*)-7-dodecenyl, and (*Z*)-9 tetradecenyl acetate (Z5-10:OAc, Z7-12:OAc, and Z9-14:OAc) [37–39]. The biosynthesis of this type of pheromone involves desaturation of palmitic acid (16C), a product of the ubiquitous fatty acid synthase machinery. The unsaturated fatty acid undergoes chain shortening, reduction, and acetylation [38]. Populations of *A. segetum* from different geographic areas differ in the ratio of their pheromone components [40, 41]. For instance the Swedish population has a ratio of Z9-14:OAc/Z7-12:OAc/Z5-10:OAc = 29/59/12 whereas the Zimbabwean population has a ratio of 2/20/78 [37]. This shift in ratios could be due to differences in the chain-shortening, in the FAR activity or less likely in the activity of the acetyltransferase.

An EST-library was previously constructed from the *A. segetum* pheromone gland, revealing candidate genes involved in pheromone production [42] and a ∆11 desaturase and a FAR involved in pheromone production were already characterized [9]. The EST library, however, contains only 2, 285 objects. We now report a more extensive database of candidate genes potentially involved in the *A. segetum* sex pheromone biosynthetic pathway, generated by next generation sequencing technology (NGS). We constructed transcriptome libraries from two tissues of *A. segetum*: pheromone gland (As_PG) and abdomen (As_AB). Furthermore, to test the function of several of the candidate genes we assayed them in a yeast heterologous expression system.

## Results and discussion

### Illumina sequencing, unigene assembly, and analysis of transcripts

**Fig. 1:**
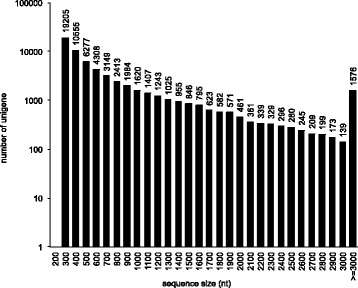
Length distribution of unigenes. The consensus sequence lengths ranging from 200 bp to more than 3,000 bp, and above each column is indicated the number of genes of each length range. The most abundant unigenes are 300 bp (19205) and the least abundant unigenes were 3000 bp (139); sequences over 3000 bp were grouped together. The number of sequences decreased as the length increased

**Fig. 2:**
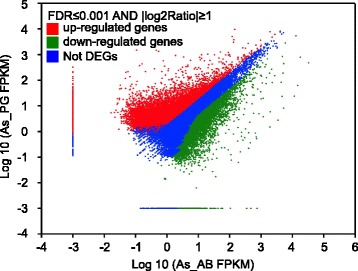
Differentially expressed unigenes displayed by FPKM in As_PG versus As_AB, in log_10_ scale. X-axis (As_AB) and Y-axis (As_PG) shows the logarithm value of normalized expression of each gene in FPKM (Fragments per kb per Million fragments). There are 21,965 unigenes that are up regulated (red dots), measured by As_PG(FPKM)/As_AB(FPKM) > 2. On the contrary, 14,292 unigenes are down regulated (green dots), since their As_PG(FPKM)/As_AB(FPKM) < 0.5. Most of the unigenes, 25,895, were equally expressed (blue dots) in both tissue (0.5 < As_PG(FPKM)/As_AB(FPKM) < 2)

In total 53 million raw clean reads were obtained from each (As_PG and As_AB) library, with a total of 4.8G clean nucleotides in each (Table 1). The clean reads from the two libraries were assembled into 62,165 consensus contigs (Table 2), including 22,633 distinct clusters (referred to as CL) and 39,532 distinct singletons (referred to as Unigene). These consensus contigs have a mean length of 733 nt, and N50 [43] =1,150, total length 45 Mb. Size distributions of the unigenes can be seen in Fig. 1 and their differential expression in the two tissues is displayed in Fig. 2. This transcriptome shotgun assembly project has been deposited at DDBJ/EMBL/GenBank under the accession GBCW00000000. The version described in this paper is the first version, GBCW01000000.

**Table 1:** Output statistics of sequencing

Sample	Total raw reads	Total clean reads	Total clean nucleotides	Q20 %	*N* %	GC %
As_PG	63,086,832	53,235,252	4,791,172,680	96.61 %	0.01 %	47.54 %
As_AB	62,193,108	54,156,050	4,874,044,500	96.62 %	0.01 %	47.44 %

Q20 percentage is proportion of nucleotides with quality value larger than 20 in reads (sequencing error rate = 1 %); N percentage is proportion of unknown nucleotides in clean reads. GC percentage is proportion of guanidine and cytosine nucleotides among total nucleotides

**Table 2:** Statistics of assembly quality

Sample	Total length (nt)	Mean length (nt)	N50	Total consensus sequences	Distinct contigs	Distinct singletons
As_PG	37,824,354	574	907	65,840	17,534	48,306
As_AB	36,392,640	618	1007	58,847	13,055	45,792
All	45,545,001	733	1150	62,165	22,633	39,532

N50 value defined as the length for which the collection of all sequences of that length or longer contains at least half of the sum of the lengths of all sequences

**Fig. 3:**
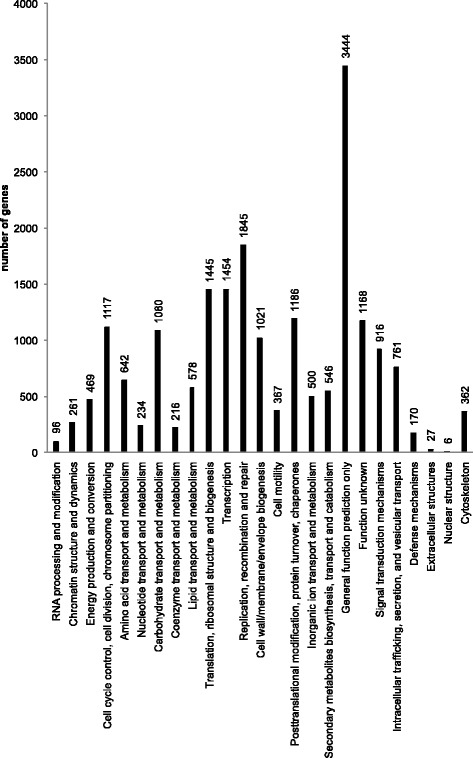
COG classification of unigenes. Histogram of COG classifications of assembled consensus sequences. Results are presented for the 25 main COG categories. The number above the column indicates number of unigenes in each category

After alignment by blastx to protein databases NCBI Nr, Swiss-Prot, KEGG and COG (e-value < 0.00001), and alignment by blastn to nucleotide database NCBI Nt, annotations were retrieved from the highest sequence similarity with the given unigenes along with their protein functional annotations. About half of the unigenes have a hit in one or more of the databases (Table 3). 14,108 unigenes hit the first record in the Nr database with *Bombyx mori* (Table 4). *A. ipsilon* got 293 hits, but this does not mean the *A. segetum* is closer to *B. mori* than *A. ipsilon*, it is just because the *B. mori* got more records deposited in GenBank than *A. ipsilon*. In terms of clusters of orthologous groups (COG), 19,911 unigenes were classed in one or more of the 26 COG functional categories (Fig. 3).

**Table 3:** Summary of annotation results

Nr	Nt	SwissProt	KEGG	COG	GO
27,344	20,403	19,143	17,351	8,502	12,049

Unigenes were annotated with the databases of Nr, Nt, Swiss-Prot, KEGG, COG and GO. Then counted the number of unigenes annotated with each database. The result is summarized as the follow table

**Table 4:** Nr annotations of assembled *A. segetum* consensus sequences

Species	Gene numbers	Percentage
*Bombyx mori*	14,108	50.56 %
*Danaus plexippus*	7,807	27.98 %
*Tribolium castaneum*	642	2.3 %
*Papilio xuthus*	569	2.04 %
*Helicoverpa armigera*	420	1.51 %
*Agrotis ipsilon*	293	1.05 %
Other	4063	14.56 %

The first column shows the species with the highest number of similar genes in descending order, the second column indicates the number of these annotated genes, and the last column shows the percentage of genes with respect to the total annotated genes sequences

**Fig. 4:**
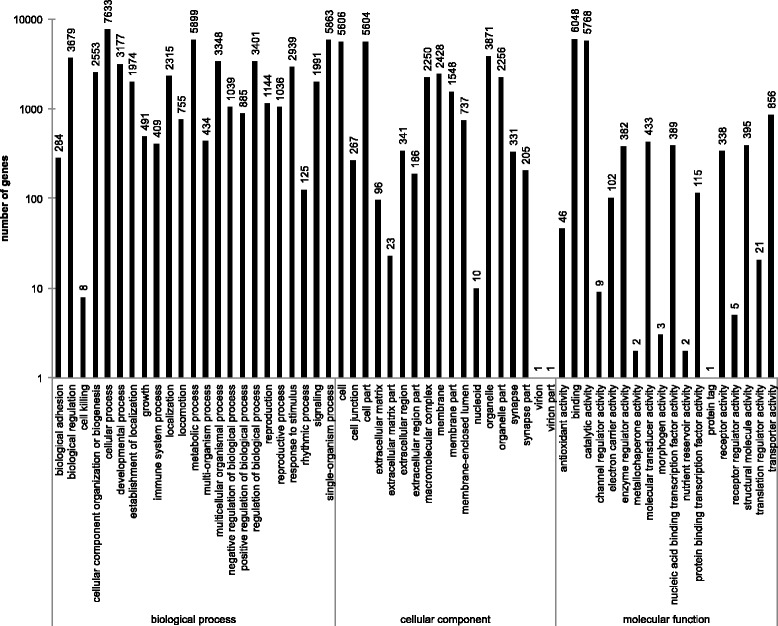
GO classification of unigenes. Histogram of GO classifications of consensus sequences. Results are summarized for the three main GO categories: biological process, cellular component and molecular function. The number on the bars represents the total number of unigenes in each category

By running the Blast2GO program [44] using Nr annotation, 11,246 (18 %) unigenes were assigned to one or more GO categories. The GO terms cellular process, binding, catalytic activity, and metabolic process were the most abundantly represented categories (over 5,000 unigenes, details see Fig. 4). These numbers and percentages are similar to the results that Gu and coauthors [45] presented from *A. ipsilon*.

**Fig. 5:**
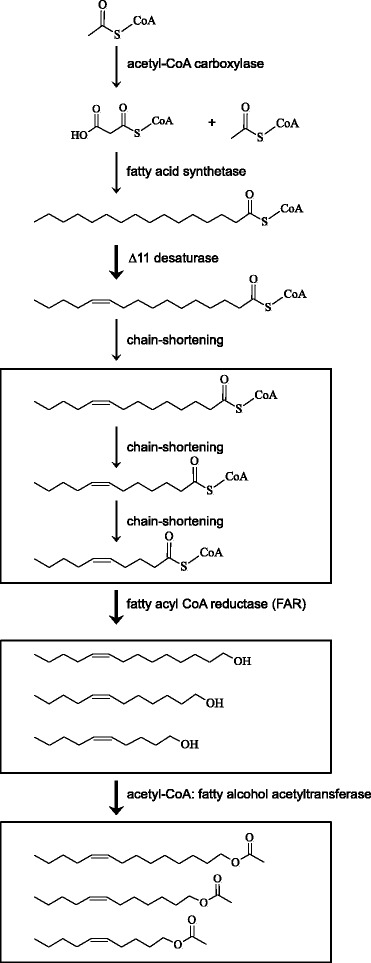
Biosynthetic pathway leading to the sex pheromone of *Agrotis segetum*, modified from [37]. It starts with carboxylation of acetyl-CoA to malonyl-CoA, and then they are entered to a cycle of fatty acid synthesis and end up with common fatty acids stearate and palmitate. The ∆11 desaturase inserts a double bond in the acyl chain and then the unsaturated fatty acid is subjected to three rounds of chain-shortening by β-oxidation, forming three acyl-chains different by two carbon atoms. These acyl-chains are then reduced by fatty-acyl CoA reductase (FAR) to make fatty alcohols, which are then acetylated to acetate esters, the final *A. segetum* pheromones. Thick arrows represent steps are functionally assayed in heterologous systems

The biosynthetic pathway (Fig. 5) leading to the pheromone components of *A. segetum* is similar to what has been reported for many other moth species [37, 45]. The key players among enzymes involved or postulated to be involved are desaturases, β-oxidation enzymes, fatty-acyl reductases, and acetyltransferases. Pheromone biosynthesis is reported to be under control by a pheromone biosynthesis activating neuropeptide (PBAN) [46]. In the following we present candidate genes related to each step in the biosynthetic pathway, their expression levels and their functional assay in yeast heterologous expression systems.

### Pheromone biosynthesis activating neuropeptide (PBAN) receptor

PBAN is released from the subesophageal ganglion (located near the brain) and is transported through hemolymph (or via the ventral nerve cord) to the pheromone gland. Upon binding to the PBAN receptor present on the pheromone gland cell membrane [47], it will induce the opening of calcium channels causing influx of extracellular calcium [7]. Then the calcium binds to calmodulin, that stimulates phosphatase (and/or kinase), which subsequently activates the FAR in the case of *Bombyx mori* [48] or in other cases the acetyl-CoA carboxylase [49] or the acetyltransferase [50] maybe regulated. In As_PG, we found one gene, Ase_17579 [GenBank: KJ622075], which is 97 % identical to *Helicoverpa zea* PBAN receptor. Its expression level is similar in As_PG and As_AB, 15.6 FPKM and 16.9 FPKM, respectively.

### Acetyl-CoA carboxylase

The rate-limiting step in fatty acid biosynthesis [51] is the ATP-dependent carboxylation of acetyl-CoA to malonyl-CoA catalyzed by acetyl-CoA carboxylase (ACCase) [52], the first step in saturated long chain fatty acid biosynthesis. ACCase is a large protein with multiple catalytic activities, working coordinately and providing malonyl-CoA substrate for the biosynthesis of fatty acids [50]. In *A. segetum*, we found one contig Ase_7442 (KJ622074), encoding the full-length of this protein. It shares 69 % amino acid identity with the ACCase of *Tribolium castaneum* and 88 % aa identity with *B. mori* ACCase. The expression level of Ase_7442 is not significantly different in As_AB (37 FPKM) and in As_PG (26 FPKM).

### Fatty acid synthase

Fatty acid synthase (FAS) is a multifunctional protein [52] that produces saturated fatty acids using malonyl-CoA and acetyl-CoA as substrate and that requires NADPH as reducing agent, in a cyclic process in which an acetyl primer undergoes a series of decarboxylative condensations with several malonyl moieties [53]. The resulting products are palmitic and stearic acid in insects, as proven by labeling studies [49, 53, 54]. We found six unigenes (KJ622068-KJ622073) that are homologous to the Aip_FAS_JX989151 [45]. In total, these unigenes are seven times more expressed (significant) in As_PG than in As_AB (FPKM 67.1 / FPKM 9).

### Desaturases

**Fig. 6:**
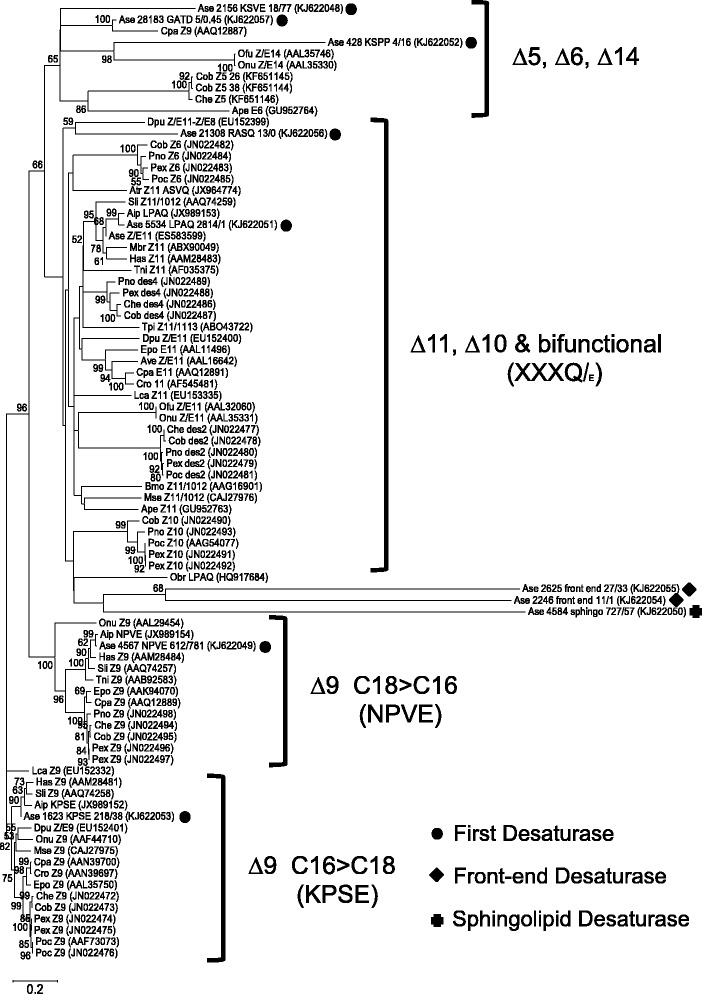
The neighbor-joining tree of selected lepidopteran desaturase genes, constructed using amino-acid sequences. Desaturases described in this study are indicated by different shapes (with signature motif displayed for the First Desaturase), followed by unigene expression levels in the gland and abdomen library, respectively (As_PG_FPKM /As_AB_FPKM). Desaturases in previous studies are named as follows: biochemical activities (if known) are indicated in connection to the species name, followed by accession number in parenthesis. Most of the desaturases used in here are First Desaturases that introduce double bond into saturated fatty acids. Among the First Desaturase, four distinctive groups formed that separate their biological functions. The ∆9 desaturases are usually used for normal fatty acid metabolism, with the “KPSE” group having preference on C16 and “NPVE” group mainly modifying C18. The ∆11,∆10 and bifunctional desaturases with the “xxxQ” motif (with a few exceptions having “xxxE” motif) exclusively involved in pheromone biosynthesis. The ∆5,∆6,∆14 group contain a mixture of different signature motifs derived from the ∆9 and ∆11 groups, and their biological function are also diverged. The tree was rooted on the ∆9-desaturase-KPSE (C16 > C18) functional class

**Fig. 7:**
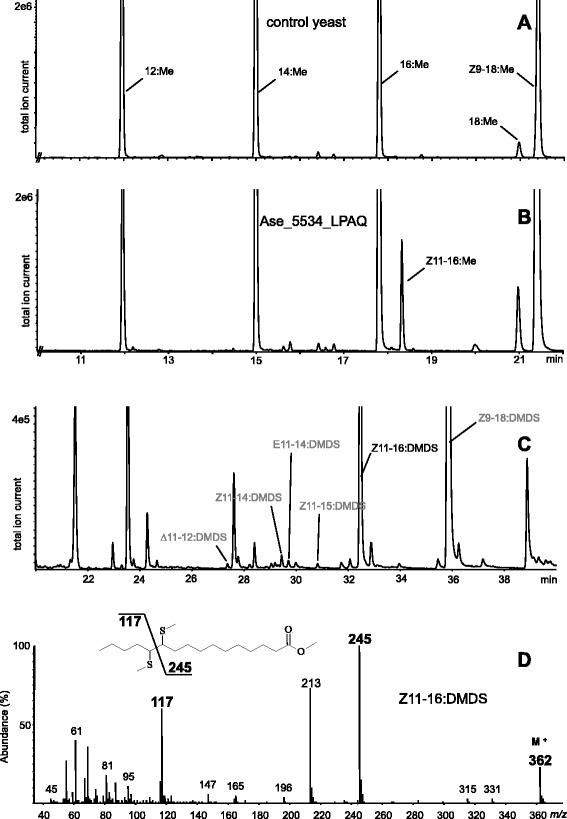
Functional characterization of the highest expressed desaturase gene, Ase_5534, in yeast expression system. GC-MS analyses of methanolyzed lipid extracts from yeast transformed with empty plasmid pYEX-CHT (**a**) and pYEX-CHT-Ase_5534 (**b**). Double bond position of the unsaturated palmitate was confirmed by DMDS derivatization (**c, d**)

Fatty acid desaturases catalyze the introduction of double bonds into acyl chains with strict regioselectivity and stereoselectivity, and can be divided into four categories [55]: 1) first desaturases, inserting a double bond into the saturated acyl chain; 2) front-end desaturases, introducing a double bond between an existing double bond and the carboxylic end; 3) omega desaturases, inserting a double bond between an existing double bond and the methyl end; 4) sphingolipid desaturases, introducing double bonds into sphingolipids which are important components of eukaryotic plasma membranes. We found 10 desaturase candidates from the two tissues, and they belong to the first desaturase (7/10), the front-end desaturase (2/10), and the sphingolipid desaturase (1/10) subfamilies. Among the first desaturase subfamily, several groups have been recognized based on phylogenetic and functional analysis and a four-letter “signature motif” has been suggested for each of them as shown in Fig. 6. These signature motifs strongly associate with the location of double bond that the desaturase is inserting in the fatty-acyl chain, with less emphasis on the chain length selectivity: ∆9_KPSE (C16 > C18) desaturases and ∆9_NPVE (C18 > C16) desaturases that are mostly involved in fatty acid metabolism [56], and ∆11,∆10 and bifunctional desaturases with the “xxxQ” motif (with a few exceptions having “xxxE” motif) exclusively involved in pheromone biosynthesis [13, 57] (Fig. 6). The Ase_1623 and Ase_4567 have the signature motif of KPSE and NPVE, respectively, that strongly suggest their roles in ordinary metabolic pathways as ∆9 desaturases. The Ase_21308 and Ase_5534 both have “xxxQ” motif, suggesting that they represent ∆11 desaturases. The Ase_5534 is the obvious candidate for pheromone biosynthesis since its expression level in As_PG is 1–2 magnitudes higher than any other candidate, and it has a very low expression level in As_AB. The functional assay confirms that it possess the ability of creating a ∆11-double bond mainly on palmitate (Fig. 7), corroborating that Ase_5534 [GenBank:KJ622051] is the desaturase responsible for pheromone production in *A. segetum*. This result is consistent with the recent study in which this gene was expressed in different yeast strains [9] and this desaturase was as a matter of fact found already in the EST analysis reported by Strandh *et al.* [42]. This desaturase introduces a double bond with Z configuration on 16:Acyl. Besides Z11-16:Acyl, some other minor products were detected: ∆11-12:Acyl, Z11-14:Acyl, E11-14:Acyl, and Z11-15:Acyl (Fig. 7). The Ase_21308 displayed no activity in our yeast expression system. Gu *et al.* [45] found a desaturase that is very close to our Ase_5534 (as shown in Fig. 6) and which should be the one involved in pheromone biosynthesis in *A. ipsilon*. Compared to [42, 45, 58], our dataset includes a larger set of desaturases isolated from the moth pheromone gland and surrounding tissues.

### β-oxidation enzymes

After the palmitate has undergone ∆11 desaturation in the *A. segetum* pheromone gland, it is subject to limited chain shortening by β-oxidation, resulting in three homologous fatty-acyl pheromone precursors with 14C, 12C, and 10C chain length (Fig. 5). Chain-shortening by β-oxidation is the action of a series of enzymes, working sequentially and forming a reaction spiral.

In the first reaction, acyl-CoA is converted to *E*2-enoyl-CoA, by ***acyl-CoA oxidases*** (in peroxisomes) and ***acyl-CoA dehydrogenases*** (in mitochondria). Four acyl-CoA dehydrogenases with different chain length specificities cooperate to assure that the complete degradation of all fatty acids with different chain length. The names of the four dehydrogenases, short-chain, medium-chain, long-chain, and very long-chain acyl-CoA dehydrogenases, reflect their chain-length specificities. Short-chain acyl-CoA dehydrogenase only acts on short-chain substrates like butyryl-CoA and hexanoyl-CoA. Medium-chain acyl-CoA dehydrogenase is most active with substrates from hexanoyl-CoA to dodecanoyl-CoA, whereas long-chain acyl-CoA dehydrogenase preferentially acts on octanoyl-CoA and longer-chain substrates [59]. Very-long-chain acyl-CoA dehydrogenase extends the activity spectrum to longer-chain substrates, including those having acyl chains of 22 and 24 carbon atoms [60]. We found a full spectrum of dehydrogenases and oxidases in *A. segetum* (Table 5). It is noteworthy that unigene6715 was expressed 72 times higher (386FPKM) in As_PG than in As_AB (5FPKM). In addition, we found two unigenes of isovaleryl-CoA dehydrogenase, which is specific for metabolism of branched-chain fatty acids [61].

**Table 5:** The gene candidates found in As_PG that may be involved in p-oxidation processes

genelD	GenBank accession	As PG FPKM	As AB FPKM	Swissprot-annotation	ko_definition
acyl-CoA dehydrogenase					
Unigene5337_All	KJ622076	2.49	0.93	Short-chain specific acyl-CoA dehydrogenase	butyryl-CoA dehydrogenase[EC:1.3.8.1]
Unigenel8315_All	KJ622077	4.35	3.26	Short-chain specific acyl-CoA dehydrogenase	butyryl-CoA dehydrogenase[EC:1.3.8.1]
CL3917.Contigl_All	KJ622078	8.02	14.14	Short-chain specific acyl-CoA dehydrogenase	butyryl-CoA dehydrogenase[EC:1.3.8.1]
Unigenel5571_All	KJ622079	24.39	67.70	Short/branched chain specific acyl-CoA dehydrogenase	short/branched chain acyl-CoA dehydrogenase [EC:1.3.99.12]
CL 1315 .Contig2_All	KJ622080	29.09	40.69	Probable medium-chain specific acyl-CoA dehydrogenase	acyl-CoAdehydrogenase [EC: 1.3.99.3]
Unigene4168_All	KJ622081	4.04	3.43	Acyl-CoA dehydrogenase family member 9	Acyl-CoA dehydrogenase family member 9 [EC:1.3.99.-]
CL252.Contigl_All	KJ622082	10.03	7.93	Very long-chain specific acyl-CoA dehydrogenase	Very long chain acyl-CoA dehydrogenase [EC:1.3.99.-]
CL622.Contig4_All	KJ579211	77.52	107.57	Trifunctional enzyme subunit beta	acetyl-CoAacyltransferase [EC:2.3.1.16]
Unigene7769_All	KJ622083	3.29	2.51	Isovaleryl-CoA dehydrogenase	Isovaleryl-CoA dehydrogenase [EC:1.3.8.4]
Unigene9593_All	KJ622084	8.77	9.31	Isovaleryl-CoA dehydrogenase	Isovaleryl-CoA dehydrogenase [EC:1.3.8.4]
acyl-CoA oxidase					
CL3960.Contigl_All	KJ622085	16.52	17.33	Probable peroxisomal acyl-CoA oxidase	acyl-CoA oxidase [EC:1.3.3.6]
CL4327.Contigl_All	KJ622086	3.85	4.30	Peroxisomal acyl-CoA oxidase	acyl-CoA oxidase [EC:1.3.3.6]
CL6898.Contigl_All	KJ622087	5.75	11.79	Probable peroxisomal acyl-CoA oxidase 1	acyl-CoA oxidase [EC:1.3.3.6]
CL7062.Contigl_All	KJ622088	14.54	18.14	Peroxisomal acyl-CoA oxidase 3	acyl-CoA oxidase [EC:1.3.3.6]
Unigenel 9521_All	KJ622089	29.25	23.07	Probable peroxisomal acyl-CoA oxidase 1	acyl-CoA oxidase [EC:1.3.3.6]
Unigene26088_All	KJ622090	2.29	0.68	Peroxisomal acyl-CoA oxidase 1	acyl-CoA oxidase [EC:1.3.3.6]
Unigene33961_All	KJ622091	3.46	2.66	Probable peroxisomal acyl-CoA oxidase 1	acyl-CoA oxidase [EC:1.3.3.6]
Unigene4386_All	KJ622092	1.74	1.78	Probable peroxisomal acyl-CoA oxidase 1	acyl-CoA oxidase [EC:1.3.3.6]
Unigene5059_All	KJ622093	1.92	1.13	Peroxisomal acyl-CoA oxidase 1	acyl-CoA oxidase [EC:1.3.3.6]
Unigene6715_A11	KJ622094	386.08	4.64	Probable peroxisomal acyl-CoA oxidase 1	acyl-CoA oxidase [EC:1.3.3.6]
enoyl-CoA hydratase					
CL1656.Contigl_All	KJ622095	16.69	23.69	Methylglutaconyl-CoA hydratase	Methylglutaconyl-CoA hydratase [EC:4.2.1.18]
CL2503.Contigl_All	KJ622096	8.85	1.87	Probable enoyl-CoA hydratase	enoyl-CoA hydratase [EC:4.2.1.17]
CL2595.Contigl_All	KJ622097	122.91	142.29	Trifunctional enzyme subunit alpha	enoyl-CoA hydratase [EC:4.2.1.17]
Unigenel4514_All	KJ622098	4.15	4.35	3-hydroxyisobutyryl-CoA hydrolase	3-hydroxyisobutyryl-CoA hydrolase [EC:3.1.2.4]
Unigenel7624_All	KJ622099	73.15	87.01	Enoyl-CoA hydratase	enoyl-CoA hydratase [EC:4.2.1.17]
Unigene6308_All	KJ622100	5.00	5.89	Enoyl-CoA hydratase domain-containing protein 3	enoyl-CoA hydratase [EC:4.2.1.17]
L-3-hydroxyacyl-CoA dehydrogenase					
Unigenel2153_All	KJ622101	11.64	1.60	3-hydroxyacyl-CoA dehydrogenase	3-hydroxyacyl-CoA dehydrogenase [EC1.1.1.35]
Unigenel5615_All	KJ622102	8.46	8.38	3-hydroxyacyl-CoA dehydrogenase	3-hydroxyacyl-CoA dehydrogenase [EC1.1.1.35]
Unigenel7583_All	KJ622103	174.80	83.79	Peroxisomal multifunctional enzyme	3-hydroxyacyl-CoA dehydrogenase [EC1.1.1.35]
Unigenel9554_All	KJ622104	103.82	78.74	Flydroxyacyl-CoA dehydrogenase	3-hydroxyacyl-CoA dehydrogenase [EC1.1.1.35]
Unigene8215_All	KJ622105	8.18	4.22	Probable 3-hydroxyacyl-CoA dehydrogenase	3-hydroxyacyl-CoA dehydrogenase [EC1.1.1.35]
3-ketoacyl-CoA thiolase					
CL2371.Contigl_All	KJ579207	10.01	5.43	3-ketoacyl-CoA thiolase	acetyl-CoA acyltransferase 2 [EC:2.3.1.16]
Unigene21478_A11	KJ622106	4.47	0.05	3-ketoacyl-CoA thiolase	acetyl-CoA acyltransferase 2 [EC:2.3.1.16]
Unigene26739_All	KJ622107	3.32	0.00	3-ketoacyl-CoA thiolase	acetyl-CoA acyltransferase 2 [EC:2.3.1.16]
Unigene28250_All	KJ622108	122.89	42.24	Trifunctional enzyme subunit beta	acetyl-CoA acyltransferase [EC:2.3.1.16]
Unigene30699_All	KJ622109	1.19	0.76	3-ketoacyl-CoA thiolase	acetyl-CoA acyltransferase 2 [EC:2.3.1.16]
Unigene5444_All	KJ622110	2.35	15.28	3-ketoacyl-CoA thiolase	acetyl-CoA acyltransferase 2 [EC:2.3.1.16]
A3,A2-trans-enoyl-CoA isomerase					
CL3311.Contigl_All	KJ622111	24.40	35.52	Enoyl-CoA delta isomerase 1	3,2-trans-enoyl-CoAisomerase, mitochondrial [EC:5.3.3.8]
Unigene2565_All	KJ622112	3.56	1.88	Enoyl-CoA delta isomerase 2	peroxisomal 3,2-trans-enoyl-CoA isomerase [EC:5.3.3.8]
Δ3,5 Δ2,4-dienoyl-CoA isomerase					
Unigenel6713_All	KJ622113	11.43	18.26	Delta(3,5)-Delta(2,4)-dienoy 1-Co A is omerase	delta(3,5)-Delta(2,4)-dienoyl-CoA isomerase [EC:5.3.3.-]

The second step of β-oxidation E2-enoyl-CoA is reversibly hydrated by ***enoyl-CoA hydratase*** to L-3-hydroxyacyl-CoA. Two categories of enoyl-CoA hydratases have been identified in mitochondria [62]. One is specialized for crotonyl-CoA (4C). The second one is long-chain enoyl-CoA hydratase, effectively hydrates medium-chain and long-chain substrates. Long-chain enoyl-CoA hydratase is a component enzyme of the trifunctional β-oxidation complex, which additionally exhibits long-chain activities of L-3-hydroxyacyl-CoA dehydrogenase and 3-ketoacyl-CoA thiolase [63], and resides in the inner mitochondrial membrane. We found six enoyl-CoA hydratases from *A. segetum* (Table 5), with similar expression level in both As_PG and As_AB.

The third reaction is the reversible dehydrogenation of L-3-hydroxyacyl-CoA to 3-ketoacyl-CoA catalyzed by ***L-3-hydroxyacyl-CoA dehydrogenase***. Four categories of L-3-hydroxyacyl-CoA dehydrogenases have been identified in mitochondria. Long chain L-3-hydroxyacyl-CoA dehydrogenase is a component enzyme of the trifunctional β-oxidation [63], which is most active with long-chain substrates. Medium-chain and short-chain L-3-hydroxyacyl-CoA dehydrogenase, are both soluble matrix enzymes, processing medium- and short-chain substrates. These three enzymes complement each other and thus assure high rates of dehydrogenation over the whole spectrum of β-oxidation intermediates [63]. In *A. segetum* this group of enzymes is represented by five unigenes (Table 5), with similar expression level in both tissues.

The final step in which 3-ketoacyl-CoA is cleaved by ***thiolase*** between its α and β carbon atoms, makes the substrate two carbons shorter. Three classes of thiolase exist in motochondria: acetoacetyl-CoA thiolase or acetyl-CoA acetyltransferase (specific for acetoacetyl-CoA), 3-ketoacyl-CoA thiolase or acetyl-CoA acyltransferase (act on C4-C16), and long-chain 3-ketoacyl-CoA thiolase that is a component enzyme of the membrane-bond trifunctional β-oxidation complex, whereas the first two thiolases are soluble matrix enzymes [62]. We found six unigenes of 3-ketoacyl-CoA thiolase from *A. segetum* (Table 5), and all of them expressed at similar level in As_PG and As_AB.

The degradation of unsaturated fatty acids requires ***auxiliary enzymes*** like ∆3,∆2-enoyl-CoA isomerase and 2,4-dienoyl-CoA reductase to modify the structure of double bonds during the β-oxidation process to ensure a continuous flow of intermediates through the β-oxidation spiral [64]. We found two unigenes of the ∆3,∆2-enoyl-CoA isomerase, one mitochondrial type and one peroxisomal. In addition, we found a ∆3,5∆2,4-dienoyl-CoA isomerase that is specialized for processing odd-numbered double bonds [61].

Insects in general have the ability to shorten long-chain fatty acids to a specific shorter chain length [65]. Jurenka [5, 66] suggests that this kind of limited chain shortening takes place in the peroxisomes. Unlike the β-oxidation in mitochondria in which substrates are thoroughly degraded into two-carbon units, the β-oxidation in peroxisomes ceases at the formation of medium-chain fatty-acyl-CoAs, because acyl-CoA oxidase is inactive toward substrates having acyl moieties of eight or fewer carbon atoms [66]. Moth pheromone components are commonly 12C and 14C, suggesting that there is still something specific about the chain-shortening being involved in moth pheromone biosynthesis.

In the present study we found representatives of all the key players and auxiliary enzymes of β-oxidation, and some of them are very high in expression level and differentially expressed among the As_PG and As_AB (Table 5), forming promising candidates to tackle the role of β-oxidation on pheromone biosynthesis either by heterologous expression or by RNAi.

### Fatty-acyl reductases

**Fig. 8:**
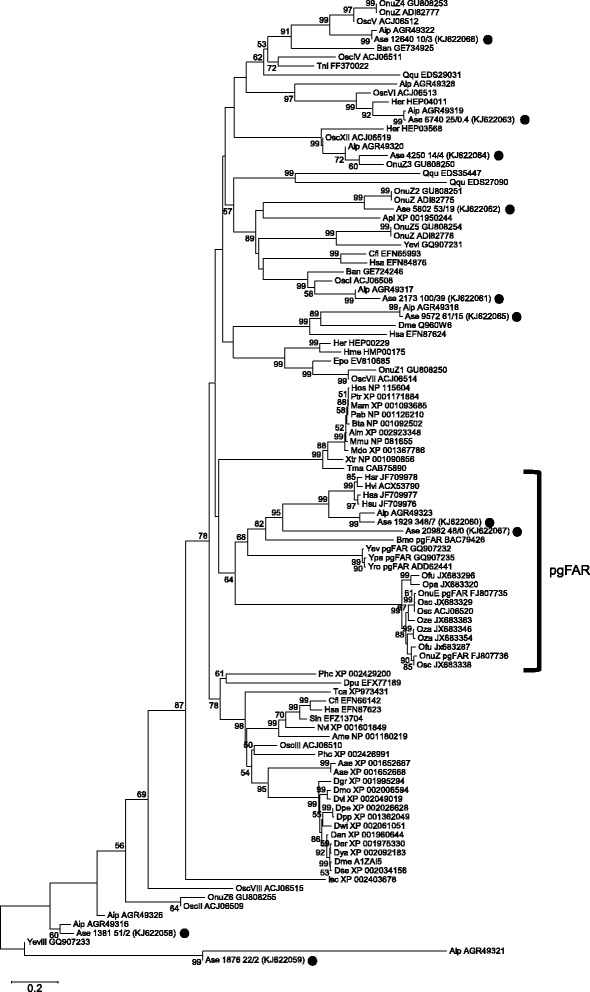
Phylogenetic relationship of FARs from arthropods, mammals and lepidoptera constructed using amino acid sequences. The pgFAR clade is marked by a black bracket, which contains previously studied functional FARs involved in moth pheromone biosynthesis. FARs identified in this study are displayed by black dots, with As_PG_FPKM and As_AB_FPKM indicated

**Fig. 9:**
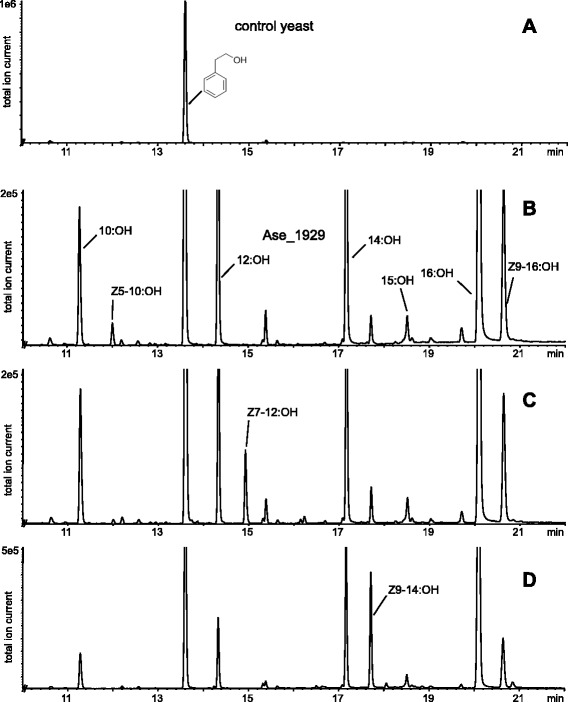
Functional assay of the highest expressed FAR in As_PG, Ase_1929 identified in this study. GC trace of hexane extract of yeast transformed by empty plasmid (**a**) and pYES2_CL1929 supplemented with Z5-10:Me (**b**), Z7-12:Me (**c**), Z9-14:Me (**d**). The control yeast produced no fatty alcohols whereas the yeast expressing Ase_1929 convert a series of fatty acids into their corresponding fatty alcohols (**b-d**)

Chain-shortened fatty-acyl precursors are reduced to the corresponding alcohols by Fatty-Acyl Reductases (FARs) [20]. FARs have been cloned and characterized from several moth species [21–23] since the first one was found in *B. mori* [20]. We found ten full-length unigenes in both the As_PG and As_AB (Fig. 8) and two of them cluster within the *pgFAR* clade [25]. We expressed the two ORFs that belong to the pheromone-producing clade in our yeast expression system. The results showed that it is the one named Ase_1929 that is responsible for reducing all the three fatty-acyl precursors (Z5-10:CoA, Z7-12:CoA, Z9-14:CoA) into their corresponding fatty alcohols (Fig. 9), and it is the most abundant transcript among all these ten unigenes (Fig. 8). This result is consistent with previous findings that a single FAR takes a wide range of the fatty-acyl substrates and convert them into fatty alcohols [24, 25, 67]. Our results complement the findings of Hagström *et al.* [9], who presented the Ase_1929 with longer chain acyl substrates (16C) and found it to be very active on them. But what appears in Fig. 9 as if the Ase_1929 when expressed in yeast is more active towards longer chain length substrates than towards shorter ones, does not necessarily represent its actual activity in the pheromone gland. This difference could partly be due to the volatility of the substrates and products, since shorter chain methyl esters and alcohols are more volatile and thus may escape from the yeast expression system during incubation. The other unigene Ase_20982 which also clustered with the *pgFAR* clade, did not show any function in our heterologous expression system. The FAR [GenBank: JX989146, protein ID: AGR49323] found by Gu *et al.* [45] clustered very close to our Ase_1929, suggesting that it is likely involved in pheromone biosynthesis in *A. ipsilon*.

### Acetyltransferases

**Fig. 10:**
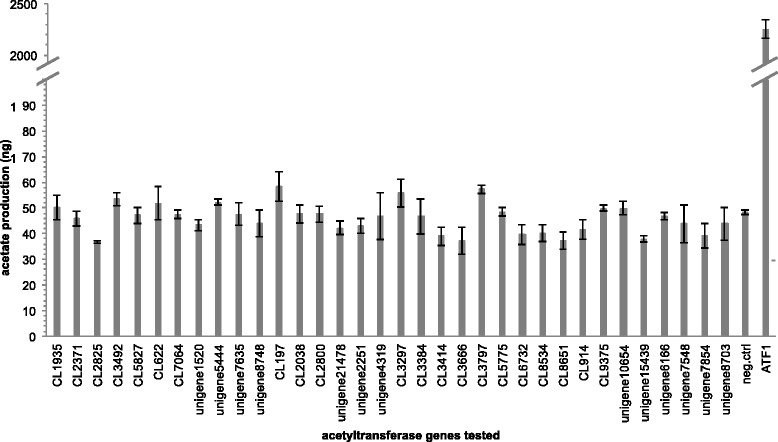
Functional assay of putative acetyltransferase genes. Y-axis represents the total amount of acetate esters (sum of Z5-10:OAc, Z7-12:OAc, and Z9-14:OAc) produced by the yeast cells transformed with candidate genes (±95 % confidence interval, *n* = 3). Negative control is yeast cell (∆*ATF1*) transformed with empty vector and the yeast strain overexpressing ATF1 gene serves as positive control. None of the 34 candidate genes produces significantly higher amount of acetate esters compared to the negative control (overlapping 95 % confidence intervals), whereas the ATF1 produces a 45-fold increase in acetate production

The genes involved in acetylation of fatty pheromone alcohols have not been cloned from any insect species. Acetyltransferases probably belong to a huge family of acyl CoA-utilizing enzymes whose products include a variety of chemicals, such as neurotransmitters [68], plant volatile esters [69, 70], constitutive defense compounds, waxes [71], phytoalexins, lignin, phenolics, alkaloids, and anthocyanins [72–74], which makes it very difficult to make functional predictions from primary sequence information alone. Attempts have been made but ended up with getting other member of the family [75]. This step is the last step in the *A. segetum* pheromone biosynthetic pathway. Most likely it does not significantly influence the ratio between the homologous acetate pheromone components [32, 33], although in several moth species the Z isomers are produced faster than the E isomers and yield more products, when the alcohol substrates were supplied to the pheromone gland homogenate [34]. Previously, a number of acetyltransferases that were cloned from plant species were studied. For example, the kiwi alcohol acetyltransferase AT9 produced butyl acetate and butyl propionate with highest catalytic activities when tested with short chain alcohols [69]. Another example is the apple alcohol acyltransferases (MpAAT1) that use coenzyme A (CoA) donors together with alcohol acceptors as substrates, which were cloned and characterized [76]. The MpAAT1 recombinant enzyme can utilize a range of alcohol substrates from short to medium straight chain (C3-C10), branched chain, aromatic and terpene alcohols. The enzyme can also utilize a range of short to medium chain CoAs [76], but alcohols longer than C10 have not been tested. Similar work was done with enzymes forming volatiles esters from banana, melon, and strawberry. The recombinant enzymes were capable of producing esters from a wide range of alcohols and acyl-CoA [70, 77, 78]. Overall, these enzymes are members of the BAHD [73, 74], generally recognized by their active site motif (HXXXD) and a conserved region (DFGWG) with likely structural significance [69]. A tBLASTn search with current published BAHDs as query against our *A. segetum* transcriptome got no hit, suggesting this moth may not express this gene family in its pheromone gland or they have undergone substantial evolutionary changes. But according to previously published putative acetyltransferase [45, 58] and the annotation results we found 34 candidates in our transcriptome (Table 6) and we heterologously expressed them in our yeast system. The ORF of each of these putative acetyltransferases was thus cloned into a yeast expression vector, pYES-DEST52, under the control of galactose inducible promoter. The ∆*ATF1* yeast strain (knock out strain that lacking most of the acetylation activity) was transformed with an individual construct and incubated in liquid culture with the culture medium supplemented with a mixture of Z9-14:OH, Z7-12:OH and Z5-10:OH. After incubation for 2 days, the total lipids were extracted and analyzed by GC-MS for acetate esters. The results did not reveal any of insect candidate genes being capable of esterifying fatty alcohols into acetate esters (Fig. 10), whereas the strain overexpressing *ATF1* was highly active (positive control).

**Table 6:** List of tested acetyltransferases that are generated by annotations and by Blastx of As_PG library with previously published ([54, 55] and references therein) promising candidates as queries

Gene object	Accession code	bp	As PG FPKM	As AB FPKM	Nr annotation	KO/COG/GO/Swissprot annotation	Species	Accession number	% Identity
CL1935	KJ579206	1542	53.53	70.39	Acetyl-CoA acetyltransferase	aeetyl-CoA C-aeetyltransferase [EC:2.3.1.9]	*Danaus plexippus*	EHJ68573	82
CL2371	KJ579207	1203	14.63	8.51	Thiolase 4	aeetyl-CoAacyltransferase 2 [EC:2.3.1.16]	*Heliothis virescens*	AGG55002	92
CL2825	KJ579208	771	6.38	14.16	Hypothetical protein KGM 16,501	Predicted acetyltransferases and hydrolases	*Danaus plexippus*	EHJ72951	87
CL3492	KJ579209	2094	16.48	10.62	Crooked neck protein	peptide alpha-N-acetyltransferase [EC:2.3.1.88]	*Aedes aegypti*	XP001653815	83
CL5827	KJ579210	1608	682.84	198.28	Sterol carrier protein 2/3- oxoacyl-CoA thiolase	Sterol carrier protein 2 [EC:2.3.1.176]	*Spodoptera littoralis*	AAT72922	94
CL622	KJ579211	1425	146.13	113.81	Fatty acid beta-oxidation complex subunit beta	acetyl-CoA acyltransferase [EC:2.3.1.16]	*Heliothis virescens*	ADB57045	95
CL7064	KJ579212	543	76.93	186.14	Acetyltransferase	Peptide alpha-N-acetyltransferase [EC:2.3.1.88]	*Agrotis ipsilon*	AGQ45625	100
Unigenel520	KJ579213	1191	136.8	89.85	Thiolase 1	aeetyl-CoA acyltransferase 2 [EC:2.3.1.16]	*Heliothis virescens*	AGG54999	93
Unigene5444	KJ579214	1113	2.35	15.28	Thiolase 3	aeetyl-CoA acyltransferase 2 [EC:2.3.1.16]	*Heliothis virescens*	AGG55001	80
Unigene7635	KJ579215	891	23.37	46.32	Palmitoyl-protein thioesterase 1 like isoform XI	Predicted acetyltransferases and hydrolases	*Bombyx mori*	XP004931556	73
Unigene8748	KJ579216	549	13.51	9.31	Dynactin 4 protein	Carbonic anhydrases/acetyltransferases, isoleucine patch superfamily	*Bombyx mori*	NP001040117	99
CL197	KJ579217	1080	20.88	20.02	Monoacylglycerol acyl transferase	2-acylglycerol O-acyltransferase 2 [EC:2.3.1.22]	*Manduca sexta*	AHH25136	72
CL2038	KJ579218	1443	5.54	30.82	Transmembrane protein nessy	lysophospholipid acyltransferase 5 [EC:2.3.1.23 2.3.1.-]	*Bombyx mori*	XP004933932	76
CL2800	KJ579219	1638	20.71	27.68	Dihydroxyacetone phosphate acyltransferase	glyceronephosphate O-acyltransferase [EC:2.3.1.42]	*Bombyx mori*	XP004921835	64
Unigene21478	KJ579220	540	4.47	0.05	Thiolase 2	aeetyl-CoA acyltransferase 2 [EC:2.3.1.16]	*Heliothis virescens*	AGG55000	73
Unigene2251	KJ579221	819	1.54	0.17	Acetyltransferase	lysophosphatidate acyltransferase [EC:2.3.1.51]	*Agrotis ipsilon*	AGQ45623	97
Unigene4319	KJ579222	1152	7.58	11.83	Acyltransferase AGPAT6	lysophosphatidic acid acyltransferase/[EC:2.3.1.51]	*Heliothis virescens*	AGG54998	96
CL3297	KJ579223	1296	12.72	12.03	Endophilin-A-like isoform XI	Endophilin-A	*Bombyx mori*	XP004929694	86
CL3384	KJ579224	1269	69.16	84.67	Ipase 1-like	Predicted hydrolases or acyltransferases	*Bombyx mori*	XP004927335	57
CL3414	KJ579225	1428	7.08	16.56	Sterol O-acyltransferase 1	Sterol O-aeyltransferase [EC:2.3.1.26]	*Danaus plexippus*	EHJ66395	66
CL3666	KJ579226	1272	38.77	53.18	Putative glycerol-3-phosphate acyltransferase	type I keratin, acidic	*Danaus plexippus*	EHJ77802	56
CL3797	KJ579227	1164	10.06	4.91	Hypothetical protein KGM19212	[EC:2.3.1.-]	*Danaus plexippus*	EHJ67359	73
CL5775	KJ579228	1179	22.36	14.42	l-acylglycerol-3-phosphate O- acyltransferase ABHD5	abhydrolase domain-containing protein 5 [EC:2.3.1.51]	*Bombyx mori*	XP004927229	85
CL6732	KJ579229	1071	7.31	8.66	Epoxide hydrolase 4-like protein	Soluble epoxide hydrolase [EC:3.3.2.10]	*Heliconius erato*	AGC92732	66
CL8534	KJ579230	1410	157.51	175.16	Dihydrolipoamide succinyltransferase component of 2-oxoglutarate dehydrogenase, partial	2-oxoglutarate dehydrogenase E2 component (dihydrolipoamide succinyltransferase) [EC:2.3.1.61]	*Papilio polytes*	BAM20532	93
CL8651	KJ579231	675	99.26	87.58	Hypothetical protein KGM 17,353	Pyruvate dehydrogenase E2 component (dihydrolipoamide acetyltransferase) [EC:2.3.1.12]	*Danaus plexippus*	EHJ75541	75
CL914	KJ579232	1962	19.4	17.94	Carnitine o-acyltransferase	Carnitine O-palmitoyltransferase 2 [EC:2.3.1.21]	*Bombyx mori*	XP004929482	75
CL9375	KJ579233	2073	16.36	9.35	Juvenile hormone epoxide hydrolase-like protein 3	Predicted hydrolases or acyltransferases	*Bombyx mori*	NP001159619	60
Unigenel0654	KJ579234	1440	10.41	5.11	Lipoamide acyltransferase	2-oxoisovalerate dehydrogenase E2 component (dihydrolipoyl transacylase) [EC:2.3.1.168]	*Bombyx mori*	XP004926652	71
Unigenel5439	KJ579235	924	0.58	6.57	Serine hydrolase-like protein 2- like	[EC:3.1.-.-]	*Bombyx mori*	XP004926488	55
Unigene6166	KJ579236	1446	5.56	27.08	Glycerol-3-phosphate acyltransferase 4	Glycerol-3-phosphate O-acyltransferase 3/4 [EC:2.3.1.15]	*Bombyx mori*	XP004925117	84
Unigene7548	KJ579237	996	15.04	13.97	Probable serine hydrolase-like	Predicted hydrolases or acyltransferases (alpha/beta hydrolase superfamily)	*Bombyx mori*	XP004924867	70
Unigene7854	KJ579238	522	3.92	1.09	acyltransferase AGPAT5	Lysophosphatidate acyltransferase [EC:2.3.1.51]	*Heliothis virescens*	AGG54997	83
Unigene8703	KJ579239	1200	3.71	3.53	Lysophospholipid acyltransferase 1-like	Lysophospholipid acyltransferase 1/2 [EC:2.3.1.51 2.3.1.-]	*Bombyx mori*	XP004927037	74

## Conclusions

We explored the data obtained from massive sequencing of pheromone producing tissue of the turnip moth and compared the expression levels of candidate unigenes with their expression levels in the abdominal epidermal tissue. This allowed identification of key parts involved in the pheromone biosynthesis pathway such as: β-oxidation enzymes, a desaturase, a fatty-acyl reductase, putative acetyltransferases, and other components involved in the fatty-acid metabolism, like acetyl-CoA carboxylase and fatty-acid synthetase. By phylogenetic analyses of desaturases and FARs, we found the most promising candidates for each gene and confirmed their function in pheromone biosynthesis. The ∆11-desaturase is a specialist interacting preferentially with 16:CoA and producing Z11-16:CoA. The FAR we found is a generalist that can reduce a broad range of saturated and unsaturated acyl substrates from 10C to 16C. We had specifically hoped to be able to clone and characterize an acetyltransferase involved in pheromone biosynthesis that has been postulated in many studies. We tested 34 genes that were annotated to be acetyltransferases in our yeast expression system (which can successfully express plant-derived acetyltransferase [10]) but it turned out that none of them was functional in comparison to the *ATF1* positive control. The nature of the acetyltransferase involved in moth pheromone biosynthesis remains illusive. In addition, we generated a list of β-oxidation enzymes, ACC and FAS that can be further tested either by heterologous expression or by RNAi.

## Methods

### Insects and tissue collection

*Agrotis segetum* were obtained from a laboratory culture, continuously maintained for more than 20 years in Lund, but repeatedly rejuvenated by addition of field-collected insects. The larvae were reared on a semisynthetic bean-based diet [79] and kept at 25 °C under a 16 h:8 h light: dark cycle. Pupae were separated by sex and placed in different jars. The third day after they emerged, 30 pheromone glands [58, 80] and abdominal epidermal tissue [6] from 3 individuals of female moths were dissected 3–4 h into scotophase [81] and stored in −80 °C freezer until RNA extraction.

### RNA extraction

Total RNA from pheromone gland and abdominal tissue were extracted using the TRIzol reagent (Life Technologies, Lidingö, Sweden) according to the manufacturer’s instructions except for three additional ethanol washes before dissolving RNA in water. RNA concentration and purity were checked on NanoDrop2000 (Thermo Scientific, Saveen Werner, Malmö, Sweden).

### Illumina sequencing and bioinformatic analysis

Twenty μg of total RNA from each sample were sent to BGI (Hong Kong Co., Ltd) for library construction, Illumina sequencing and subsequent bioinformatic analysis.

Reads were assembled using Trinity [82] into contigs. Then the reads are mapped back to contigs, get sequences without Ns and cannot be extended on either end. Such sequences are defined as unigenes. Then TGICL [83] is used to assemble all the unigenes from As_PG and As_AB to form a single set of non-redundant unigenes. Then gene family clustering was performed and unigenes were divided to two classes. One is clusters, which the prefix is CL and the cluster id is behind. In one cluster, there are several unigenes which similarity between them is more than 70 %. The other is singletons, which the prefix is unigene. Unigene sequences are aligned with blastdb using blastx (E-value < 0.00001). Sequence orientations are determined according to the best hit in the database. Bioinformatic data were viewed and further processed using Geneious version (6.1.6), created by Biomatters and available from http://www.geneious.com.

The calculation of unigene expression was performed using the FPKM method (Fragments Per kb per Million fragments) [84, 85], the formula is FPKM = 10^6^C/NL/10^3^. Set FPKM to be the expression of unigene A, and C to be number of fragments that uniquely aligned to unigene A, N to be total number of fragments that uniquely aligned to all unigenes, and L to be the base number in the CDS of unigene A. The FPKM method eliminates the influence of different gene lengths and sequencing levels on the calculation of gene expression. Therefore the calculated gene expression can be directly used for comparing the differences in gene expression between samples [85].

Functional annotations of unigenes were conducted, based on protein sequence similarity, towards the KEGG Pathway, COG [86] and Gene Ontoloty (GO) databases. Briefly, we search all unigene sequences against protein databases (NR, SwissProt, KEGG, COG) using blastx (E-value < 0.00001). Based on NR annotation, we use Blast2GO program [44] to get GO annotation of all unigenes. After getting GO annotation for every unigene, we use WEGO software [87] to do GO functional classification for all unigenes.

### Phylogenetic reconstruction

Sequences used for phylogenetic reconstructions were retrieved from the GenBank (http://www.ncbi.nlm.nih.gov) database. Neighbor-Joining trees were constructed using Mega version 4.0 [88]. Briefly, multiple sequence alignments were run using the MAFFT (online version) and the output FASTA format data were plugged into Mega software. Genetic distance model was JTT, 1500 replicates, Neighbor-Joining as tree building method.

### Fatty acid precursors

Z9-18:Me, 16:Me were purchased from Larodan Fine Chemicals AB (Malmö, Sweden). Z5-10:OH, Z7-12:OH, Z9-14:OH were purchased from Pherobank (Wageningen, The Netherlands, 98 % purity). Z9-14:Me, Z7-12:Me, Z5-10:Me were prepared from their corresponding alcohols by previously described methods [89]. All FAMEs and OH were dissolved in 96 % ethanol in a 0.1 M stock solution. All alcohols used as reference compounds were from our laboratory collection of pheromone compounds.

### Construction of expression vector for functional assay

For the construction of yeast expression vectors containing the candidate genes, specific primers with attB1 and attB2 sites incorporated were designed for amplifying the ORF of genes of interest. The PCR products were subjected to agarose gel electrophoresis and purified using the Wizard® SV Gel and PCR Clean up system (Promega Biotech AB, Nacka, Sweden). The ORFs were subcloned into the pDONR221 vector in presence of BP clonase (Life Technologies), after confirmation by sequencing, the correct entry clones were selected and do LR reaction with pYES2-DEST52 (for FARs and acetyltransferases) or pYEX-CHT-DEST vector (for desaturases), and resulting expression clones were analyzed by sequencing.

### Functional assay in yeast

The resultant recombinant expression vectors harboring the candidate genes were introduced into the INVSc (MATa HIS3 LEU2 trp1-289 ura3-52) (for FARs), the ∆*ATF1* knockout strain (for acetyltransferase), or the double deficient ole1 elo1 strain (MATa elo1::HIS3 ole1::LEU2 ade2 his3 leu2 ura3) (for desaturase) of the yeast *Saccharomyces cerevisiae* [19] using the S.c. easy yeast transformation kit (Life Technologies). For selection of uracil (and leucine) prototrophs, the transformed yeast was allowed to grow on SC plate containing 0.7 % YNB (w/o aa, with Ammonium sulfate) and a complete drop-out medium lacking uracil (and leucine) (Formedium™ LTD, Norwich, England), 2 % glucose, 1 % tergitol (type Nonidet NP-40, Sigma-Aldrich Sweden AB, Stockholm, Sweden), 0.01 % adenine (Sigma) and containing 0.5 mM oleic acid (Sigma) as extra fatty acid source. After 2 days (7 days for ole1 elo1 strain) at 30 °C, individual colonies were picked up to inoculate 10 mL selective medium at 30 °C and grown at 300 rpm for 48 h. Yeast cultures were diluted to an OD600 of 0.4 in 10 mL fresh selective medium containing 2 % galactose (2 mM CuSO_4_) with supplementation of a biosynthetic precursor. Each FAME or fatty alcohol precursor was prepared at a concentration of 100 mM in 96 % ethanol and added to reach a final concentration of 0.5 mM in the culture medium [19]. In the acetyltransferase assay the yeasts were supplemented with a mixture of the three alcohols Z9-14:OH, Z7-12:OH and Z5-10:OH. Yeasts were cultured in 30 °C in a shaking incubator at 30 °C.

### Fatty acid/alcohol/acetate analysis

After 48 h of incubation yeast cells were harvested by centrifugation at 3,000 rpm. For the analysis of desaturase products, total lipids were extracted using 3.75 mL of methanol/chloroform (2:1, v/v), in a glass tube. One mL of HAc (0.15 M) and 1.25 mL of water were added to the tube to wash the chloroform phase. Tubes were vortexed vigorously and centrifuged at 2000 rpm for 2 min. The bottom chloroform phase, about 1 mL, containing the total lipids, were transferred to a new glass tube. Fatty acid methylesters (FAMEs) were made from this total lipid extract. The solution of total lipids was evaporated to dryness under gentle nitrogen flow. One mL of sulfuric acid (2 % in methanol) was added to the tube, which was then vortexed vigorously and incubated at 90 °C for an hour. After incubation, 1 mL of water was added, mixed well, and then 1 mL of hexane was used to extract the FAMEs [10].

Fatty alcohols and acetates were extracted from cells using 800 μL of hexane plus sonication. After brief centrifugation, the supernatant was transferred to a new tube and subjected to GC-MS analysis.

Double bond positions were confirmed by dimethyl disulfide (DMDS) derivatization [13], followed by GC-MS analysis. FAMEs (50 μL) were transferred to a new tube and 50 μL DMDS was added and incubated at 40 °C overnight, in the presence of 5 μL of iodine (5 % in diethyl ether) as catalyst. Hexane (200 μL) was added to the sample and the reaction was neutralized by addition of 50-100 μL Na_2_S_2_O_3_ (5 % in water). The organic phase was recovered and concentrated under a gentle nitrogen stream to 40-50 μL.

### Gas chromatography - mass spectrometry (GC-MS)

The methylesters, fatty alcohols and acetates were subjected to GC-MS analyses on a Hewlett Packard 6890 GC coupled to a mass spectrometer HP 5973. The GC was equipped with an INNOWax column (30 m × 0.25 mm i.d. × 0.25 μm film thickness, Agilent Technologies), and helium was used as carrier gas (average velocity: 33 cm/s). The MS was operated in electron impact mode (70 eV), scaning between *m/*z 30 and *m/*z 400, and the injector was configured in splitless mode at 220 °C. The oven temperature was set to 80 °C for 1 min, then increased at a rate of 10 °C/min up to 210 °C, followed by a hold at 210 °C for 15 min, and then increased at a rate of 10 °C/min up to 230 °C followed by a hold at 230 °C for 20 min.

DMDS derivatives were analyzed on an Agilent 6890 GC system equipped with HP-5MS capillary column (30 m × 0.25 mm i.d. × 0.25 μm film thickness, Agilent Technologies) coupled with an HP 5973 mass spectrometer. The oven temperature was set at 80 °C for 1 min, raised to 140 °C at a rate of 20 °C/min, then to 250 °C at a rate of 4 °C/min and held for 20 min [11].

Data were analyzed using the ChemStation software (Agilent, Technologies, USA).

### Accession code

PBANr: KJ622075

ACC: KJ622074

FAS: KJ622068–KJ622073

Desaturases: KJ622048–KJ622057

β-oxidation enzymes: KJ622076–KJ622113

FARs: KJ622058–KJ622067

Acetyltransferases: KJ579206–KJ579239

## Abbreviations

ACC: Acetyl-CoA carboxylase
FAS: fatty acid synthase
FAR: fatty-acyl reductase
GC-MS: gas chromatography mass spectrometry
PCR: polymerase chain reaction
Z9-14:OAc: (*Z*)-9-tetradecenyl acetate
Z7-12:OAc: (*Z*)-7-dodecenyl acetate
Z5-10:OAc: (*Z*)-5-decenyl acetate
Z9-14:OH: (*Z*)-9-tetradecenol
Z7-12:OH: (*Z*)-7-dodecenol
Z5-10:OH: (*Z*)-5-decenol
Z9-14:Me: (*Z*)-9-tetradecenoic methyl ester
Z7-12:Me: (*Z*)-7-dodecenoic methyl ester
Z5-10:Me: (*Z*)-5-decenoic methyl ester
